# [Corrigendum] Endophytic fungi from mangrove inhibit lung cancer cell growth and angiogenesis *in vitro*

**DOI:** 10.3892/or.2023.8608

**Published:** 2023-07-31

**Authors:** Xin Liu, Xin Wu, Yuefan Ma, Wenzhang Zhang, Liang Hu, Xiaowei Feng, Xiangyong Li, Xudong Tang

Oncol Rep 37: 1793–1803, 2017; DOI: 10.3892/or.2017.5366

Subsequently to the publication of the above article, the authors have drawn to the attention of the Editorial Office that a few inadvertent errors were made during the assembly of Fig. 6 on p. 1802. In the first instance, the images selected to represent the A549 cell line in Fig. 6A were inadvertently shown as the data for the NCI-H460 cell line in Fig. 6C and vice versa, and so the data shown for Fig. 6A and C have been interchanged in the revised version of this figure. Moreover, the representative image for panel ‘3’ in Fig. 6A of the above article (and so, now panel ‘3’ in Fig. 6C of the corrected version) was wrongly copied across from that of panel ‘2’ in Fig. 6C (now, panel ‘2’ in Fig. 6A of the corrected version).

The authors were able to re-examine their original data files, and realize how the errors were made during the assembly of this figure. The revised version of Fig. 6, with the data originally shown in Fig. 6A and C now interchanged, also showing the correct data for panel ‘3’ in Fig. 6C, is shown on the next page. Note that the errors made in assembling this figure did not affect the overall conclusions reported in the paper. The authors are grateful to the Editor of *Oncology Reports* for allowing them the opportunity to publish this Corrigendum, and all the authors agree with its publication. They also apologize to the readership for any inconvenience caused.

## Figures and Tables

**Figure 6.**
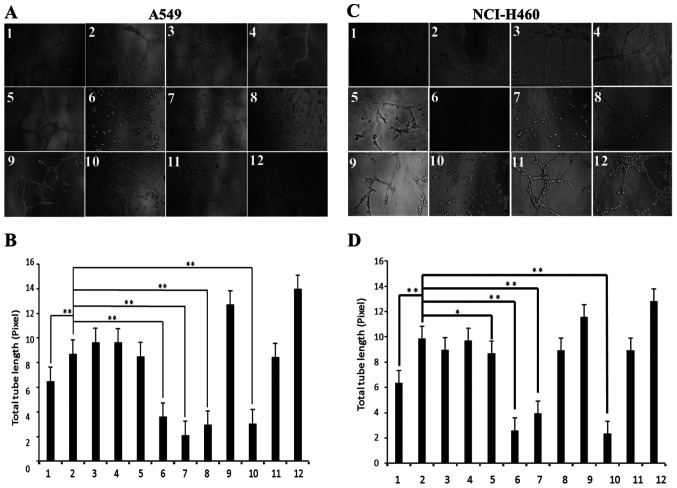
Effects of endophytic fungal fermentation on HPV-16 E7-stimulated lung cancer angiogenesis *in vitro.* HUVECs were incubated at 37°C for 6–8 h in the conditioned media derived from HPV-16 E7-transfected A549 or NCI-H460 cells treated with different culture broths of endophytic fungi (zj-2, zj-9, zj-12, zj-14, zj-17, zj-23, zj-25, zj-36, zj-67 and zj-70). (A and C) Tube formation was observed under a phase-contrast microscope (magnification, ×200). (B and D) The total tube length in three random view-fields/well was determined using Scion image software, and the average value was calculated. Lane 1, empty vector transfection control; lane 2, HPV-16 E7 transfection control (abbreviation, E7); lane 3, zj-2 + E7; lane 4, zj-9 + E7; lane 5, zj-12 + E7; lane 6, zj-14 + E7; lane 7, zj-17 + E7; lane 8, zj-23 + E7; lane 9, zj-25 + E7; lane 10, zj-36 + E7; lane 11, zj-67 + E7; lane 12. zj-70 + E7. All data are expressed as mean ± SD of three independent experiments; *P<0.05, **P<0.01.

